# Missed opportunities for participation in prevention of mother to child transmission programmes: Simplicity of nevirapine does not necessarily lead to optimal uptake, a qualitative study

**DOI:** 10.1186/1742-6405-4-27

**Published:** 2007-11-22

**Authors:** Lungiswa L Nkonki, Tanya M Doherty, Zelee Hill, Mickey Chopra, Nikki Schaay, Carl Kendall

**Affiliations:** 1 Health Systems Research Unit, Medical Research Council, Cape Town, South Africa; 2 Research Programme, Health Systems Trust, Cape Town, South Africa; 3 Tulane School of Public Health and Tropical Medicine, Tulane University, New Orleans, USA; 4 School of Public Health, University of the Western Cape, Cape Town, South Africa

## Abstract

**Background:**

The objective of this study was to examine missed opportunities for participation in a prevention of mother-to-child transmission (PMTCT) programme in three sites in South Africa. A rapid anthropological assessment was used to collect in-depth data from 58 HIV-positive women who were enrolled in a larger cohort study to assess mother-to-child HIV transmission. Semi-structured interviews were conducted with the women in order to gain an understanding of their experiences of antenatal care and to identify missed opportunities for participation in PMTCT.

**Results:**

15 women actually missed their nevirapine not because of stigma and ignorance but because of health systems failures. Six were not tested for HIV during antenatal care. Two were tested but did not receive their results. Seven were tested and received their results, but did not receive nevirapine. Health Systems failure for these programme leakages ranged from non-availability of counselors, supplies such as HIV test kits, consent forms, health staff giving the women incorrect instructions about when to take the tablet and health staff not supplying the women with the tablet to take.

**Conclusion:**

HIV testing enables access to PMTCT interventions and should therefore be strengthened. The single dose nevirapine regimen is simple to implement but the all or nothing nature of the regimen may result in many missed opportunities. A short course dual or triple drug regimen could increase the effectiveness of PMTCT programmes.

## Introduction

HIV/AIDS is the leading cause death among young children and is estimated to account for 40% of the deaths in 2000 in South Africa [1]. The HIV prevalence amongst antenatal clients across the country is 29%[2] and projections indicate that without effective prevention of mother-to-child transmission (PMTCT), the child mortality rate is likely to have continued to rise in subsequent years [3].

The PMTCT package comprises of a series of interventions namely VCT, ARV prophylaxis (in South Africa, short course nevirapine) [4], infant feeding counselling and postnatal follow-up care. Each aspect of the programme is important and a deficiency in any of the programme aspects will impact negatively on overall effectiveness, thereby compromising the ultimate goal of PMTCT, infant HIV-free survival [5].

A single maternal dose of nevirapine (NVP) between two and twenty-four hours before delivery and an infant dose within 72 hours post-delivery reduces mother-to-child HIV transmission by up to 50% [6]. The simplicity and effectiveness of this regime has led to its widespread adoption in PMTCT programmes in developing countries. However, operational studies have found that less than half of mothers testing HIV positive routinely receive even this simple regimen [7-9]. This poor performance has been used to delay the introduction of more complex but effective prophylactic interventions such as short course AZT during pregnancy boosted by single dose NVP during delivery.

In South Africa the PMTCT programme has reached national coverage. Over 95% of pregnant women attend antenatal care (ANC) with an average of more than 3 visits per pregnancy[10] and uptake of HIV testing for pregnant women has reached 70% in areas where the programme has been prioritized [11]. Despite the high ANC and HIV testing uptake, data from a 2003 programmatic evaluation showed that up to 50% of women who are recruited into the PMTCT programme do not actually receive nevirapine according to the national protocol [12]. National routine data indicates a 51.7% national NVP coverage, with large variations between and within districts. For instance three districts within a poorly resourced province have an overall coverage of 70% whereas there is one with only 16.5%[11]. The aim of this study was to investigate why, in the context of apparently functioning and accessible health services in terms of ANC coverage, such large numbers of women are not getting a relatively simple HIV intervention.

## Methods

A rapid anthropological assessment was used to collect in-depth data from 58 HIV positive women who were enrolled in a larger cohort study to assess mother to child HIV transmission. The quantitative cohort study had a final sample of 625. 123 Women were randomly selected from the quantitative cohort using a random number table. Randomly selected women were then interviewed until we reached a point of data saturation. Data collection took place between April and June 2005. Semi-structured interviews were conducted with randomly selected women from the larger cohort in order to gain an understanding of their experiences of antenatal care and to identify missed opportunities for participation in PMTCT.

### Study sites

Three sites were purposively selected to reflect different socio-economic profiles, rural-urban locations and HIV prevalence rates. Site A is a peri-urban area with an antenatal prevalence of 15%[12], a well resourced health system and a higher socio-economic profile than the other sites. Site B, is a rural area in one of the poorest regions of South Africa with a poorly resourced health system and an antenatal HIV prevalence of 28%[12]. Site C, is a peri-urban area with an antenatal prevalence of 41% [12] and an moderately well resourced health system compared to the other two sites.

### Data collection and analysis

Selected women were approached and consented in their community and either interviewed at home or, if privacy was an issue, in the project office. Interviews lasted between 45 minutes and 3 hours and were conducted in local languages by six trained field researchers. The six field researchers used a semi-structured guide, which contained suggested questions for each research theme. Data analysed for this paper included descriptions of testing, counseling and diagnosis; details of support and care received after diagnosis (including details of the PMTCT programme); perceptions of quality of care and contextual information about diagnosis, disclosure and living conditions. Each field researcher conducted one interview a day, during which they recorded brief field notes. Each afternoon the field researcher worked with the study site supervisors to convert their field notes to English fairnotes. Fairnotes were then keyed into MS word by the study transcriber.

Preliminary data analysis occurred concurrently with data collection. We made use of the inductive generation of coding categories. In this approach, investigators first review all notes; identify important descriptive categories and themes and code sections of text for the presence of these themes. The investigators did this individually, then met and discussed the results until they reached consensus.

Ethical approval was obtained from the University of Western Cape all participants signed informed consent for interviews. The anonymity of participants was protected during data collection and analysis by the use of participant codes.

## Results

All the women had attended antenatal care during their pregnancies with the exception of one. The mean age of the mothers was 26.3 years. A quarter (15/58) of the HIV positive women reported having missed their NVP dose. Reasons for missing the dose fell into three main categories (Table 1).

**Table 1:** Reasons for missed opportunities per category

Overall Sample size n = 58	**Categories n = 15**	**Reasons for missed opportunities**
	Category 1 (n = 6)	No HIV test during ante-natal care
	Category 2 (n = 2)	Tested and did not receive results until after delivery
	Category 3 (n = 7)	Tested and did not receive results until after delivery

### 1: No HIV test during ante-natal care

Six of the fifteen women who missed their NVP belonged to this group. Five of the six women would have liked to have been tested but were not tested due to health system failures including non-availability of counselors, supplies such as HIV test kits or consent forms: Only one respondent made a conscious effort to avoid finding out her HIV status due to fear of being HIV positive (Table 2).

**Table 2:** No HIV test during ante-natal care

*Non-health systems reasons:*
'She did not go to Hospital A for ANC because she knew that she will have to test at ANC. She thought of the baby she is carrying and that if at present moment she can be told for sure that she is positive she will die for sure. PG06TM'
*Health systems reasons:*
"She was always told that a "VCT" nurse was not there on the days she visited and she was also told that there are no injections to draw blood. RG07VM"
'She was attending her ANC at <a local clinic> which she pays R10 return in a taxi to get to this clinic and it is a closer clinic to her. She was not tested during her ANC because there were not forms to sign before getting tested. RG05VM"

### 2: Tested and did not receive results until after delivery

Two respondents belonged to this group, both of whom attended ante-natal care on numerous occasions. In one case health workers failed to notice that the respondent had not received her test results and in the other case the woman was tested the day before she gave birth and was given her results the day after birth (Table 3).

**Table 3:** Tested and did not receive results until after delivery

*Health Systems reasons:*
'She says she comes close to stay at the hospital since it was her first time to get a baby but the nurses took blood from her at the last moment and not even having time to tell her good or bad things about testing and the worst thing is that she was not given NVP yet she was here for almost a month and she was treated like someone who was not attending ANC who just came in on labour at the last moment. Yet nurses are preaching that people should attend ANC so that they don't miss opportunities to know about how to be the mother for the first time and she feels it was very bad for her to realise that she was supposed to get NVP. RG06WB'

### 3: Tested, received results but did not take NVP

Seven women tested and knew their results but did not receive nevirapine at the correct time or at all. For three women the reasons were related to their personal situation: losing the tablet, forgetting to take the tablet because of an intense labor, and avoiding collecting the tablet because they did not believe their test results: For four women the reasons were directly related to health system failures including health staff giving the women incorrect instructions about when to take the tablet and health staff not supplying the women with the tablet to take (Table 4).

**Table 4:** Tested, received results but did not take NVP

*Non Health Systems reasons:*
'She said she did not come for 'environment' <Nevirapine> because she did not believe that she was HIV+. UG06TN'
'The labour went very fast and she forgot the tablet at home whilst she was rushed to hospital. When she got to the hospital, she delivered immediately whilst she was about to ask the nurse. The child became infected, sick and died...She thinks if she has used the tablet (Nevaripine) the child would still be alive now. RG01VM'
*Health Systems reasons:*
'A day after delivery the nurse came to her, asked whether she got the tablet. She told the nurse no. The nurse asked her to follow her and when they reach the room (privately) she asked whether she was told about it, she agreed. The nurse asked her why she didn't ask or remind the nurse to give her the tablet and the (nurse) blamed on her for being stupid. The nurse gave her the tablet which was whitish according to her and the nurse told her to find water somewhere and drink it when I asked her whether they told her the name of the tablets she mentioned Nevirapine. She said <Site B> had nurses that were like they were burning in hell according to her. She was not happy to be a patient at <Site B> and will never advise a person to go there.
'She's not willing to meet the nurses again. It was better if she didn't know her status. She hasn't taken her child for nine months because of that reason. RGO8WB'
'She was never given the tablet when she delivered her baby and was not told why she was not given. (She mentioned that it was written on the card that she is having "Pre – AIDS"). She was told by the nurse at a local clinic that the nurses at hospital will know that they will have to give her this tablet because it is written on the card. RG02VM'
'(On Tuesday) the doctor told her to go to the labour ward because she was about to deliver. She told the doctor that she is not feeling any labour pains. The nurse gave her the Nevirapine tablet and told her to take it immediately. Indeed she took it immediately whilst she was not feeling labour pains. She did not have labour pains until Thursday 6H30 when she felt labour pains. She was asked by another nurse if she was given Nevaripine and she told that nurse that she was given it on Tuesday and she took it immediately. That nurse said they are not going to give her another one instead they will give drops to her baby after the baby is born. RG08VM'
'The nurse gave her the tablet to take it immediately on the same day although she was not feeling labour pains. On the (next day) at about 1 am she felt labour pains and she was given another tablet. RG09VM'

## Discussion

In this study there were a series of missed opportunities that led to women not receiving nevirapine according to the national PMTCT protocol[4]. A quarter (15) of the women reported not taking nevirapine. Six of the women did not get tested and were of unknown status prior to delivery. Reasons for not testing were mainly health systems failures such as non-availability of counselors and supplies. Two of the 15 women did not receive their HIV test results until after delivery. In both instances the women had attended antenatal care on numerous occasions yet health workers failed to give them NVP in time. Seven of the 15 women tested, received results but did not take NVP not because of stigma, ignorance but because of the immediate context of the birth process in addition to health system failures.

Fear of knowing one's HIV status and disbelief of test results have been described previously as important reasons for drop out from PMTCT services [13,14] however, in our sample this explained only a minority of missed opportunities. Evidence from other programmes suggests that such fears are likely to be reduced further as the programme becomes more established[15]. Instead it was health system constraints related to testing and the provision of results that were the key reasons for missed opportunities. A lack of counselors and testing equipment was found to be prevalent across the country during early evaluations of the PMTCT pilot programme [12].

Of great importance is the functioning of the health system. 40% of our respondents had not been tested due to health systems failures. HIV testing serves as an entry point to PMTCT. A weak health system allows for leakage in essential steps such as HIV testing, which undermines the intervention. Strengthening testing uptake and logistics is urgently required. However, it is important to note that these missed opportunities occurred within the context of opt-in VCT. Routine offer (opt out) of HIV testing within antenatal care (i.e. antenatal HIV testing is part of routine screening for infections, including hepatitis B, syphilis and rubella) has been advocated. There is evidence from various studies which demonstrates that an opt-out approach to VCT identifies a greater proportion of those infected [16-20] and provides greater opportunities for HIV care and treatment.

However, the discussions on the VCT opt-out approach assume that the quality of care in all settings is optimal. Findings from developing countries do not support this assumption. For instance a recent publication from South Africa showed only 28.6% of women from a rural site had a syphilis test performed [5]. Given these results it is unclear how the VCT opt-out approach would address the current missed opportunities occurring in the VCT opt in approach.

The second reason for missed opportunities in this study was health system constraints related to mothers not receiving their results. The testing kit used in this context is the rapid HIV test. It is therefore unclear why women were not given their results and this issue requires further exploration.

The last groups of women were those who knew their status and also knew they had to take NVP but still did not take it. For reasons related to tablet provision and instruction giving, in many cases the problem stemmed from poor communication and a lack of a locus of responsibility. Improving communication could reduce these missed opportunities. On the otherhand the locus of responsibility presents a challenge. According to the PMTCT protocol women should get NVP during antenatal care to self administer at the onset of labour. However, in this group the responsibility of administering the NVP fell more with the healthcare workers. Furthermore 92% of deliveries in South Africa are attended by trained health personnel. But even in this context women failed to receive NVP [21].

The apparent simplicity of the present NVP regimen gives women only one opportunity to reduce transmission and this opportunity is too often missed. The first-line regimen suggested by WHO is either a triple or dual combination short course regimen from 32–36 weeks of pregnancy through labour and delivery and for one week postpartum to mother and infant [22]. We argue that the recently revised WHO recommendations for a more efficacious short course regimen may, despite its apparent complexity, actually reduce missed opportunities, among our study women 7 out of the 15 missed opportunities could have been averted with a multi dose regimen. If one or two doses of this short course are missed, the implications are less serious in terms of efficacy than if the single NVP dose is missed, though missing a dose in the short course may have implications for future drug resistance[22].

Evidence from studies of other diseases suggest that the more complex regimen may also result in improved adherence as studies show higher adherence with multiple doses such as daily doses instead of erratic doses such as once or twice weekly[23]. Providing a regimen that starts early in pregnancy should also be feasible as South Africa has an antenatal attendance rate of 90% and a mean number of ANC visits greater than three[10].

Whilst the 7 out of 15 women would have benefited from the complex regimen, six out of 15 women who were not tested would not have. It is clear that health system strengthening is essential for the success of any new interventions irrespective of whether the intervention is simple or not. Greater resources, management and integration with routine maternal and child health care have been recommended to reduce these shortcomings and the need for this is further highlighted by the findings of this study.

The limitations of this study were that the data was only collected from the women's point of view. Collecting data on only the women's point of view was an appropriate step given that national evaluations [8] had demonstrated quantitative missed opportunities. In fact a local evaluation[24] had cautioned against looking at each step of the programme independently for example paying attention to individual variables such as number of women who received counseling, of those who received counseling the number tested and the number that received the results. This type of analysis masks the cumulative effect of these leakages. This study has shed light on the nature of missed opportunities and health systems leakages on the continuum of care for PMTCT. These could be further explored through collecting data from the health providers. The health provider perspective will be a useful follow-up step in order to ascertain health providers understanding of the underlying reasons for the missed opportunities which arose from health systems failures.

Finally monitoring and evaluation of the quality of care even in the context of dual/triple therapy from different perspectives (Patient and Provider) will be essential in identifying barriers for PMTCT.
